# Prevalence of Neonatal Hyperphenylalaninemia in Yazd Province, Iran

**Published:** 2015-05

**Authors:** Mahtab Ordooei, Majid Jafarizadeh, Mohsen Mirzaei, Hasan Ashoori, Ali Zare, Hossein Shojaeifar

**Affiliations:** 1 Department of Pediatrics, Shahid Sadoughi University of Medical Sciences, Yazd, Iran;; 2 Children Growth Disorder Research Center, Shahid Sadoughi University of Medical Sciences, Yazd, Iran;; 3 Student Research Committee, Shahid Sadoughi University of Medical Sciences, Yazd, Iran

## Dear Editor,

Neonatal screening is a process in which infants are screened for various inborn disorders that may possibly lead to early diagnosis and the prevention of the imperative disability and distress caused later on as a result of congenital and/or inherited diseases.^1^^,^^2^ In numerous countries, the screening process lead to the detection of certain congenital diseases, such as Phenylketonuria (PKU), one of many common inborn disorders caused by a metabolic error, a result of Phenylalanine hydroxylase (PAH) deficiency.^3^ The severity of this disorder depends on the extent of enzyme deficiency and may diverge from vastly elevated concentrations (20 mg/dl) as in the classic PKU to mildly elevated levels of phenylalanine (2-20 mg/dl).^4^^,^^5^

In several parts of Iran, neonatal screening of PKU has already begun. During this descriptive cross-sectional study, the results of neonatal screening of PKU in Yazd province were surveyed and all infants born in the period of May 2010 to June 2011 were included in this study. After parental consent, blood samples were collected from the heels of newborns, three days after birth by special filter papers and delivered to Shahid Akbari clinic (the provincial center of the newborn screening laboratory in Yazd).****Screening was performed in three steps; first, Phenylalanine (Phe) levels were determined using the Guthrie test that is an enzymatic colorimetric method (PKU kit-Misagh Talashgaran, Iran). Phe concentrations higher than 2 mg/dL were contemplated as possible PKU cases and required re-examination. In the second step, the newborns suspected to PKU were tested for Phe again using the enzymatic colorimetric method. To confirm the final diagnosis, blood samples of newborns with Phe levels ≥2 mg/dl recorded in the second step were tested by high performance liquid chromatography (HPLC) (ClinRep®, Germany). Cases with high levels of Phe (≥2 in HPLC) confirmed as hyperphenylalaninemia.

During the one-year study period, a total of 22,131 newborns went through the screening process. Four cases were diagnosed as PKU patients, giving a prevalence of 1/5532 (0.018%). One of the four infants (25%) had a Phe level of above 20 mg/dl (classic PKU) and three infants (75%) had serum phenylalanine levels between 2 and 20 mg/dl, showing mild hyperphenylalaninemia. 

Neonatal screening programs for PKU have been implemented in Yazd province since 2009. The current study shows the annual incidence of PKU was estimated to be 1/5532 in Yazd. This means that, in comparison with other countries, Yazd has a high incidence of hyperphenylalaninemia alone. It may be due to abundant consanguineous marriage in Yazd (as a traditional area in the center of Iran).^6^ High incidence of this disorder in the region makes a comprehensive screening program necessary for the management of the PKU disease.
